# Evaluation of the INS-1 832/13 Cell Line as a Beta-Cell Based Screening System to Assess Pollutant Effects on Beta-Cell Function

**DOI:** 10.1371/journal.pone.0060030

**Published:** 2013-03-21

**Authors:** Tine L. M. Hectors, Caroline Vanparys, Anna Pereira-Fernandes, Geert A. Martens, Ronny Blust

**Affiliations:** 1 Systemic Physiological and Ecotoxicological Research, Department of Biology, University of Antwerp, Antwerp, Belgium; 2 Diabetes Research Center, Brussels Free University-VUB, Brussels, Belgium; Ecole Normale Supérieure de Lyon, France

## Abstract

Environmental pollutants have recently emerged as potential risk factors for metabolic diseases, urging systematic investigation of pollutant effects on metabolic disease processes. To enable risk assessment of these so-called metabolic disruptors the use of stable, robust and well-defined cell based screening systems has recently been encouraged. Since beta-cell (dys)functionality is central in diabetes pathophysiology, the need to develop beta-cell based pollutant screening systems is evident. In this context, the present research evaluated the strengths and weaknesses of the INS-1 832/13 pancreatic beta-cell line as diabetogenic pollutant screening system with a focus on beta-cell function. After optimization of exposure conditions, positive (exendin-4, glibenclamide) and negative (diazoxide) control compounds for acute insulin secretion responses were tested and those with the most profound effects were selected to allow potency estimations and ranking of pollutants. This was followed by a first explorative screening of acute bisphenol A and bis(2-ethylhexyl)phthalate effects. The same approach was applied for chronic exposures, focusing primarily on evaluation of acknowledged chronic stimulators (diazoxide, T0901317, exendin-4) or inhibitors (glibenclamide) of insulin secretion responses to select the most responsive ones for use as control compounds in a chronic pollutant testing framework. Our results showed that INS-1 832/13 cells responded conform previous observations regarding acute effects of control compounds on insulin secretion, while bisphenol A and bis(2-ethylhexyl)phthalate had limited acute effects. Furthermore, chronic exposure to known beta-cell reactive compounds resulted in deviating insulin secretion and insulin content profiles compared to previous reports. In conclusion, this INS-1 subclone appears to lack certain characteristics needed to respond appropriately to acute pollutant exposure or long term exposure to known beta-cell reactive compounds and thus seems to be, in our setting, inadequate as a diabetogenic pollutant screening system.

## Introduction

One of the hypotheses recently postulated with regard to the current diabetes pandemic is the “metabolic disruptor” hypothesis, referring to the involvement of environmental pollutants in the development of metabolic diseases [1]–[5]. Besides epidemiological studies linking pollutant exposure to increased diabetes prevalence [6], [7], experimental studies have shown that some widespread environmental pollutants such as persistent organic pollutants [8], bisphenol A [9], and some phthalates [10] are able to induce insulin resistance and alter pancreatic beta-cell function, the two pathophysiological hallmarks of type 2 diabetes [11], [12]. Although evidence for metabolic disruption by environmental pollutants is accumulating, a clear overview of which compounds should be considered risk factors is missing. Systematic investigation of the metabolic disruptor potency of thousands of pollutants by means of animal testing would be time consuming, very expensive and ethically questionable. Alternative approaches such as first line cell based screening for identification and prioritization of high risk pollutants are therefore highly encouraged [13]–[16]. As such, the development and evaluation of physiological relevant in vitro systems to enable metabolic disruptor screening is crucial [17].

Since pancreatic beta-cell functionality is considered to be the main determinant for the development of diabetes [18], the need to generate beta-cell based systems to screen for diabetogenic metabolic disruptors is evident. Despite the pivotal role of beta-cells, only very few reports have focused specifically on pollutant effects on beta-cell function (or mass) [6] and just one study has been published, to our knowledge, with regard to screening a broad range of pollutants using a beta-cell model [19]. Makaji et al. (2011) [19] showed that for the pollutants tested with the murine beta-TC-6 cell line, only bisphenol A, the single well described insulinotropic pollutant [9], affected beta-TC-6 function. However, although confirmation of bisphenol A-stimulated insulin secretion advocates the use of beta-TC-6 cells for pollutant screening, their physiology profoundly deviates from that of primary beta-cells. For instance, insulin secretion occurs with a left shift of the glucose dose response curve, half maximal stimulation of insulin secretion occurs at 0.5 mM glucose and maximal induction of insulin secretion at physiological high glucose comprises only a 2- to 4-fold increase [20]–[22]. Due to this restricted physiological relevance and limited dynamic range, other cell systems with higher relevance for primary beta-cell function should be considered. One of the most physiologically relevant beta-cell models currently available is the INS-1 832/13 cell line [23]. This genetically modified INS-1 cell subclone was previously selected for its robust glucose responsiveness over the physiological range of glucose concentrations (2.8 – 16.7 mM glucose) and with levels of key glucose sensing proteins (e.g. GLUT-2 and glucokinase) comparable to those of primary rodent beta-cells [23], [24]. Furthermore, they retain a differentiated cell phenotype over more than six months in culture [23], [24]. These characteristics made it a widely used tool for studying various aspects of beta-cell function [23]–[26] and are advantageous in a compound screening context.

In this study, the application of the INS-1 832/13 cell line as a beta-cell based screening model for diabetogenic pollutants was evaluated based on prior optimization of exposure conditions, followed by testing of pharmaceutical reference compounds and environmental test compounds.

## Materials and Methods

### Routine cell culture

INS-1 832/13 cells [23] were kindly provided by C. Newgard (Duke University, Durham, NC, USA) and were cultured in 10 mL complete medium composed of RPMI 1640 medium supplemented with 10% fetal bovine serum, 50 IU/mL penicillin, 50 mg/L streptomycin, 10 mM HEPES, 2 mM L-glutamine, 1 mM sodium pyruvate, and 50 µM beta-mercaptoethanol. Cells were split twice a week. Cells were grown in a 37°C incubator under a humidified atmosphere containing 5% CO_2_ and cultures were routinely verified as mycoplasma free with the PCR based Venor^™^ GeM Mycoplasma Detection kit (Sigma-Aldrich, Bornem, Belgium). All cell culture reagents were obtained at Sigma-Aldrich, except for sodium pyruvate, penicillin-streptomycin and trypsin/EDTA (Gibco, Invitrogen LT, Merelbeke, Belgium) and fetal bovine serum (Hyclone, Thermo Fisher Scientific, Erembodegem, Belgium).

### Cell treatment

All tested compounds were obtained at Sigma-Aldrich, except for DEHP which was purchased from LGC Standards (Molsheim, France). Experiments were performed in Primaria^™^ 24-well plates (BD Biosciences, Erembodegem, Belgium)**.** Acute exposures (2 h) were performed during the last phase of the insulin secretion assay. For chronic exposures cells were treated with compounds for 72 h in complete medium, followed by the insulin secretion assay in the absence of the compounds. A detailed outline of the final experimental set up for compound exposures is discussed in the text.

### Insulin secretion assay

Cells were washed twice with 300 µL glucose-free Krebs-Ringer Bicarbonate buffer (KRB) (116 mM NaCl, 1.8 mM CaCl_2_·2(H_2_O), 0.8 mM MgSO_4_·7(H_2_O), 5.4 mM KCl, 1 mM NaH_2_PO_4_·2(H_2_O), 26 mM NaHCO_3_, and 0.5% BSA, pH 7.4) followed by preincubation for 1 h at 37°C in 500 µL glucose-free KRB. Thereafter, cells were washed two times with 300 µL glucose-free KRB, prior to a 2 h incubation in 500 µL KRB under low (2.8 mM) and high (16.7 mM) glucose and the listed test conditions (Table 1). After 2 h, medium was collected and stored at −20°C. For chronic experiments, samples of the culture medium were taken prior to the insulin secretion assay to assess insulin secretion over a 24 h period. To extract total insulin content, cells exposed to 2.8 mM glucose KRB were washed twice with ice-cold PBS (with Mg^2+^ and Ca^2+^) and 250 µL acid ethanol (1.5% HCl (37%), 18.5% MilliQ, 80% ethanol (95%)) was added. After three freeze/thaw cycles (−80°C /4°C), cells were scraped of the plates and centrifuged for 5 min at 2500 rpm (4°C). Supernatants were stored at −20°C until analysis. Insulin was detected using the Rat Insulin Enzyme Immunoassay Kit according to the manufacturer's instructions (Alpco Diagnostics, Salem, USA). Data were normalized using cell number, as determined by manual counting using a hemocytometer. Insulin secretion and content were expressed as ng insulin per 100,000 cells. The insulin secretion index (ISI) was calculated as the ratio of GSIS over BIS. GSIS or glucose stimulated insulin secretion is the amount of insulin secreted after stimulation with 16.7 mM glucose, BIS or basal insulin secretion is the amount of insulin secreted after 2.8 mM glucose stimulation. These glucose concentrations represent physiological extremes and are the most frequently used conditions for evaluation of compound effects on insulin secretion. All results were obtained from three independent experiments and represent the response of three cell batches at different passage numbers (range used passage numbers: 42–57, with start up at passage 32). Each cell batch was tested in duplicate, resulting in n = 6, unless otherwise stated.

**Table 1:** Selected compounds which alter insulin secretion in acute (hours) and chronic (days) exposure scenario's.

Compound	Target	Reported effect			Control	Reference	Concentrations	
		Exposure	BIS	GSIS			Standard	Higher
Diazoxide (Diaz)	K_ATP_-channel	Acute	=	↓	−	[23]	100 µM	325 µM
		Chronic	↑	↑	+	[49]–[51]		
			=	↑				
			↑	↑				
Glibenclamide (Glib)	K_ATP_-channel	Acute	↑	↑	+	[32], [52]	100 nM	100 µM
		Chronic	↓	↓	−	[53]–[54]		
			↑	↓				
T0901317 (T090)	Liver X receptor (LXR)	Acute	=	=	=	[33]	1 µM	10 µM
		Chronic	=	↑	+	[33], [45]		
			↓	↑				
			↑	↑				
Exendin-4 (Ex-4)	Glucagon-like peptide-1 receptor	Acute	=	↑	+	[31], [32], [57]	1 nM	10 nM
		Chronic	=	↑	+	[32], [58]		

Reported effects on basal (BIS) and glucose stimulated (GSIS) insulin secretion, and the primary targets via which the compounds exert their effects are given, together with the expected control function (positive (+), negative (−), no effect ( = )). Results from all types of in vitro beta-cell systems and species were considered. The most commonly used concentrations (“standard concentrations”), the higher concentrations selected and abbreviations used in this paper (between brackets), are also provided.

### Statistical analyses

**Figure 1:**
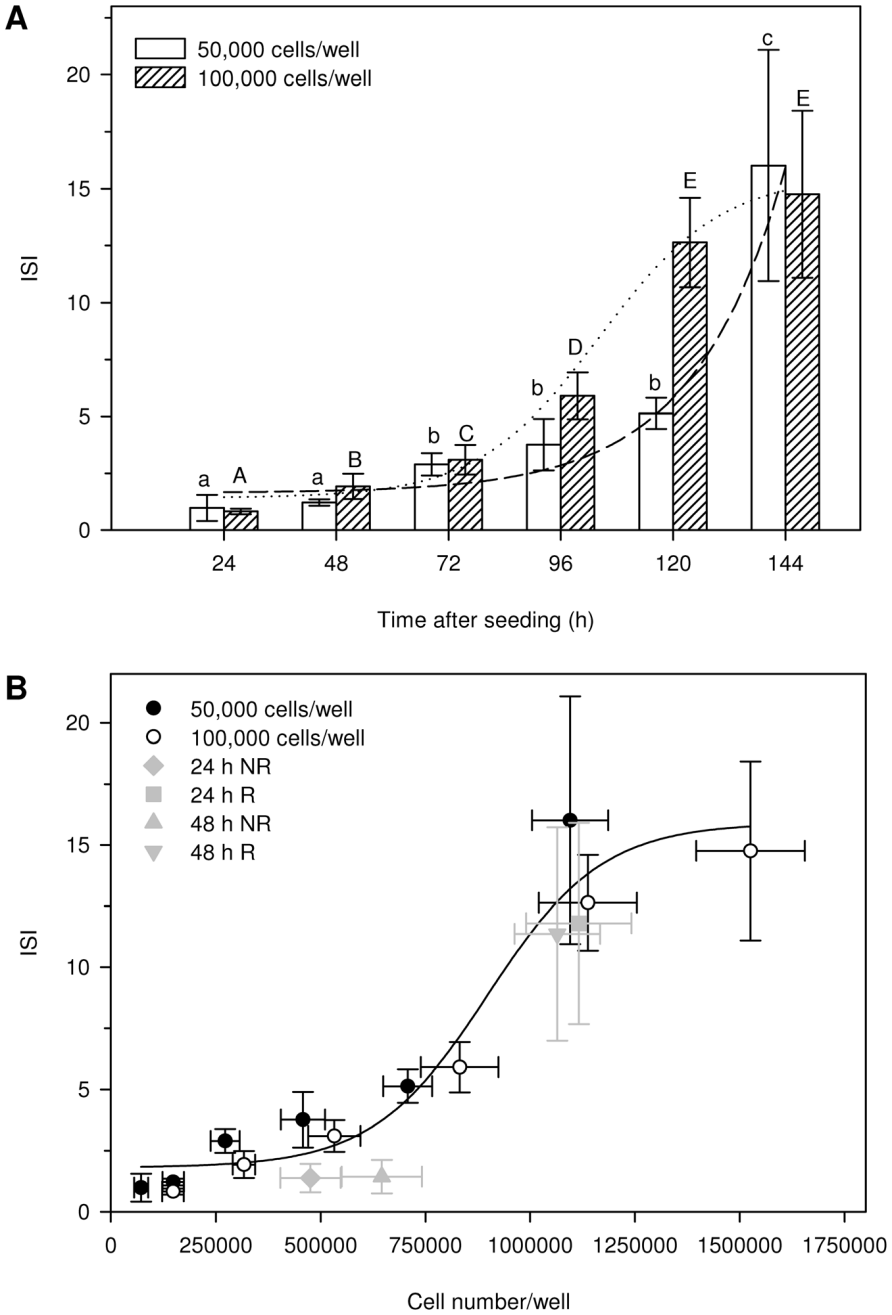
Optimization of the exposure set up in terms of robust insulin secretion responses. (A) Glucose stimulated insulin secretion expressed as insulin secretion index (ISI) (n = 6) at different time points after seeding at a density of 50,000 cells/well (white bars) and 100,000 cells/well (shaded bars). Different letters indicate significant differences in ISI between the different time points within one seeding density (Two-way ANOVA, Tukey's post-hoc, p<0.05) with lower case letters for comparisons within a seeding density of 50,000 cells/well and capital letters for a seeding density of 100,000 cells/well. Non-linear regression curves (sigmoid, 4 parameter fit; restraint maximum ISI = 15) were fitted for the 50,000 cells/well (dashed line) and 100,000 cells/well (dotted line) datasets. (B) ISI as a function of cell number per well at the moment of insulin secretion assays. Seeding density of 50,000 cells/well is indicated with black circles, 100,000 cells/well with white circles. A non-linear regression line (sigmoid, 4 parameter fit; restraint maximum ISI = 15) was fitted based on the combined 50,0000 cells/well and 100,000 cells/well seeding density datasets to determine dose response relationship between final cell number and ISI. Insulin secretion results for only 1 medium replacement 24 h (diamond) or 48 h (triangle) after seeding are referred to as NR (Non Refresh), medium replacement every 24 h, starting from 24 h (square) or 48 h (reversed triangle) after seeding is indicated as R (Refresh). Data represent the mean ± SD (n = 6 for ISI, n = 12 for cell number) of three independent experiments.

Prior to analysis, all data were tested for normality with Shapiro-Wilk statistics (W>0.90). When appropriate, data were log transformed to meet the normality assumption. The differences in ISI at the two different seeding densities in function of time and differences in insulin secretion between different treatments for both low and high glucose were tested using mixed ANOVA models. Each performed model was controlled for biological variation by adding passage number as random variable. Model selection always started with the full model and then succeeded with the removal of non-significant terms. All data are expressed as the mean (± standard deviation (SD)) response of the three passage numbers. Post-hoc Dunnett's test for comparison with control and Tukey's multiple comparison test for comparison of all groups were used in case of statistically significant treatment effects. Significance was set at p<0.05. All statistical analyses were performed using the software package SAS (SAS 9.2, SAS Institute Inc., Cary, NC, USA), except for non-linear regression of ISI data in function of cell number per well (Fig. 1) which was evaluated using the dynamic curve-fitting option of SigmaPlot (SigmaPlot 11.0, Systat Software Inc, Chicago, USA).

## Results

### Optimization of exposure conditions maximizing insulin responsiveness

To obtain a condition with robust glucose responsiveness and insulin secretion induction, insulin secretion was compared at two different seeding densities and at different time points. Cell densities were 50,000 and 100,000 cells/well in a 24-well plate, respectively 26,316 and 52,632 cells/cm^2^. Medium was replaced every 24 h. The results in Figure 1A clearly show that induction of insulin secretion follows a time-dependent increase as determined by non-linear regression analysis, both for 50,000 cell/well seeding density (R^2^ = 0.9819; p = 0.027) and 100,000 cells/well seeding density (R^2^ = 0.9933; p = 0.010). No further increase in insulin secretion is observed after reaching an ISI of approximately 14 as illustrated by absence of significant differences between ISI's obtained for 100,000 cells/well seeding density at 120 h versus 144 h after seeding. To evaluate the influence of final cell density on insulin secretion performance, ISI was expressed as a function of final cell number per well (Fig. 1B). From this graph, it is clear that final cell density at the moment insulin secretion assays are performed, non-linearily affects insulin secretion (Fig. 1B) (R^2^ = 0.9502; p<0.0001; sigmoidal, 4 parameter dose response curve). Maximal induction is reached at a final density of ± 1,250,000 cells/well (657,895 cells/cm^2^). Experimental set up should therefore be constructed in such a way that this density is reached at the moment insulin secretion assays are performed. Bearing in mind that besides acute (minutes till a few hours), also chronic (days) exposures should be feasible, growth stimulating or inhibiting effects of compounds should be anticipated. In addition, potential contact inhibition due to overloaded wells at the time of insulin secretion assessment should be avoided. Following this rationale, the time point at which secretion assays were performed in further experiments was fixed at 120 h after seeding at a density of 100,000 cells/well.

To further optimize conditions for chronic exposures, insulin secretion was assessed for different exposure set ups. The two questions addressed in these experiments were (1) what is the maximal time of exposure possible and (2) is medium refreshment necessary or not. In the set ups tested, medium was refreshed for the first time either 24 h or 48 h after seeding and renewed every 24 h or left on the cells for the remaining period. Refreshment of medium every 24 h greatly affects glucose responsiveness, independent of the timing of first replacement (data not shown). Medium renewal after seeding was essential for a significant and strong response to glucose. ISI results were 11.79 ± 4.12 and 11.36 ± 4.37 when the first medium renewal was done after 24 h or after 48 h respectively. There was no significant difference in ISI between both first refreshment time points. When plotting ISI as a function of final cell number, it is clear that refreshment stimulates cell growth, allowing cells to reach a density at which near maximal ISI is found (Fig. 1B). Refreshing the medium only once reduces cell growth to a density which is within the range of final densities which have limited insulin secretion capacity, as shown in our first experiment (Fig. 1B).

Based on these results, the optimal conditions to investigate effects on glucose stimulated insulin secretion consist of a seeding density of 100,000 cells/well (52,632 cells/cm^2^), 48 h recovery, medium (acute exposures) or treatment (chronic exposures) refreshment every 24 h and insulin secretion assays 120 h after seeding. Even for acute exposures (2 h, during insulin secretion assay) medium is renewed every 24 h, to assure maximal glucose stimulated insulin secretion capacity.

### Literature based selection of potential positive and negative control compounds

To construct a framework in which the potency of compounds to affect beta-cell function can be assessed, stable and strong positive and negative control compounds should be included. In the present research, INS-1 832/13 cells were evaluated with regard to their potential to study both short (hours) and long (days) term pollutant effects, urging selection of compounds which have been described to affect insulin secretion acutely, chronically or both. The characteristics of each of the chosen compounds, mainly pharmaceuticals, are illustrated in Table 1.

For each of the control compounds, 2 concentrations were tested, preferably based on reports of chronic exposure: a concentration reflecting the most frequently used concentration i.e. 'standard concentration', and a higher concentration i.e. 10 times higher than the standard concentration, unless use of other concentrations has been reported (e.g. glibenclamide and diazoxide). For each concentration tested, cell viability was higher than 80% compared to control in a 72 h exposure scenario, as determined by a neutral red cytotoxicity assay following the protocol described by ICVAMM (2001) [27] (data not shown).

50 µM 1-methyl-3-isobutylxanthine (IBMX), routinely used as a positive control for glucose stimulated insulin secretion in insulin secretion assays was included as extra control to verify whether general exposure conditions affected normal INS-1 832/13 responsiveness to synthetic stimuli.

### Acute effects of pharmaceutical compounds: selection of positive and negative controls for insulin secretion assays

**Figure 2:**
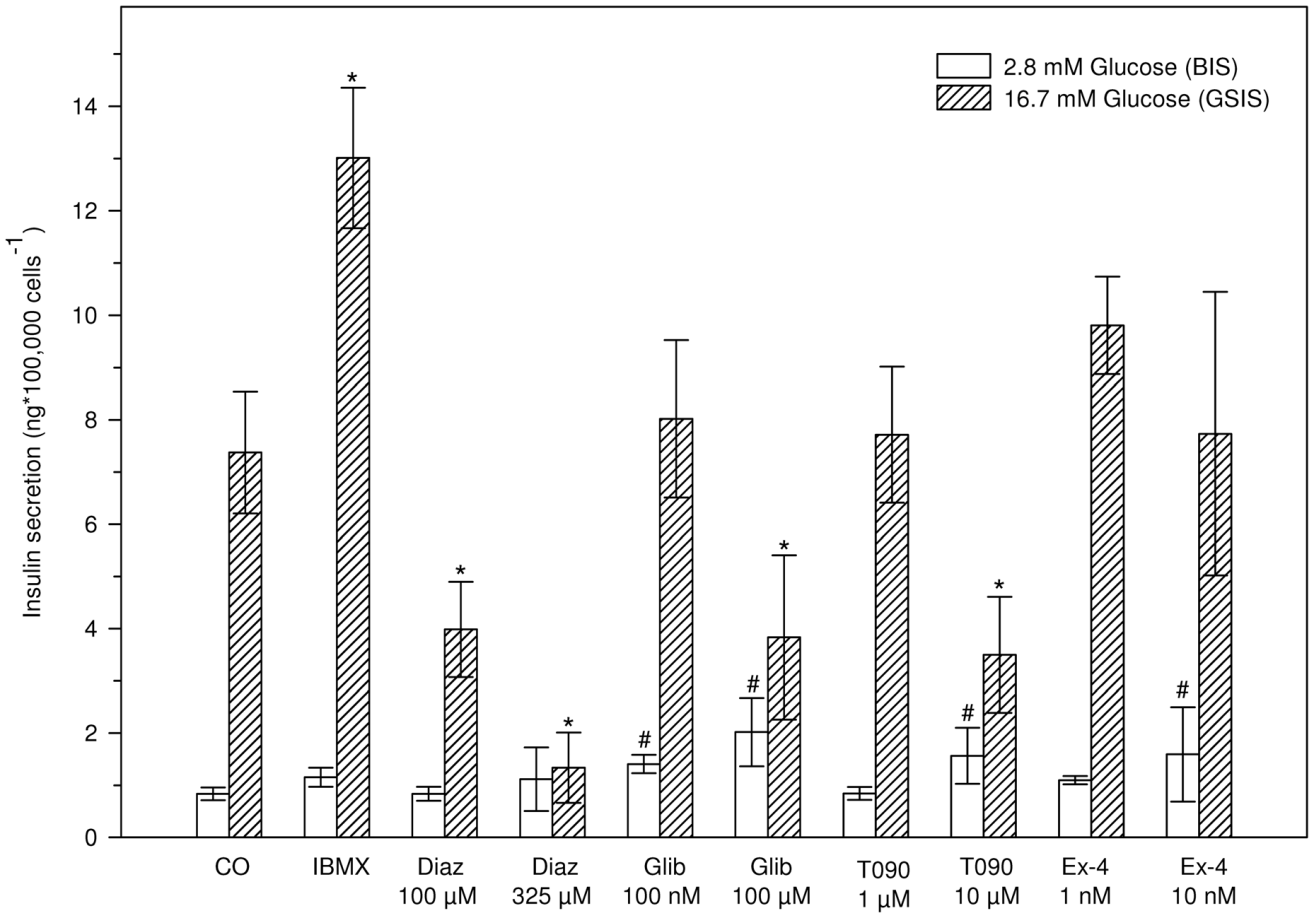
Acute effects (2 h) of selected pharmaceuticals on insulin secretion. BIS responses are represented as white bars and GSIS responses as shaded bars. Data represent the mean ± SD (n = 6) of three independent experiments. ^#^*indicates significant differences with the respective control (^#^2.8 or *16.7 mM glucose) (Two-way ANOVA, Dunnett's post-hoc, p<0.05).

To select the most potent inhibitors and activators of insulin secretion, acute effects of pharmaceuticals on BIS and GSIS were examined and results are depicted in Figure 2. IBMX (50 µM) positively stimulated insulin secretion at high glucose levels, but did not alter BIS. 100 µM and 325 µM diazoxide decreased GSIS respectively 1.85 ± 0.52 times and 5.51 ± 2.91 times compared to control. Both concentrations glibenclamide increased BIS, while 100 µM glibenclamide also significantly decreased GSIS. The same was observed for the highest concentration of T0901317 (10 µM), which increased BIS, but decreased GSIS, while 1 µM T0901217 had no effect. Exendin-4 had no effect on GSIS but at 10nM augmented BIS with 90%.

### Acute effects of pollutants: bisphenol A and bis(2-ethylhexyl)phthalate

**Figure 3:**
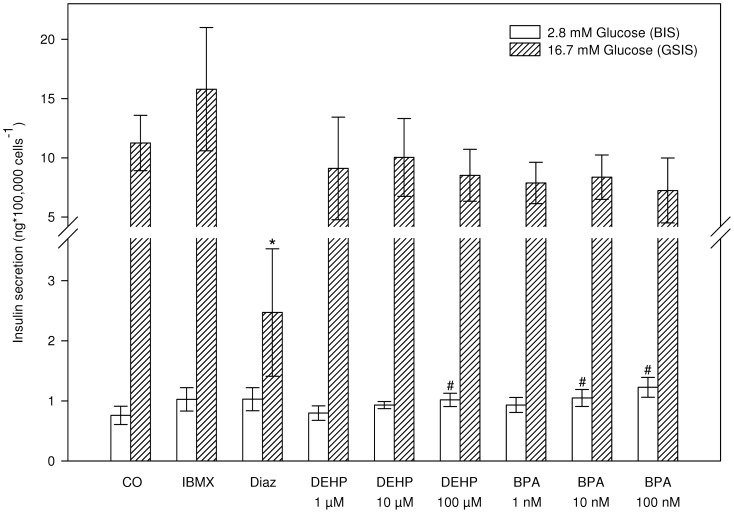
Acute effects (2 h) of the pollutants DEHP and BPA on insulin secretion. BIS responses are represented as white bars and GSIS responses as shaded bars. IBMX (50 µM) and diazoxide (Diaz) (100 µM) were added as positive and negative control, respectively. Data represent the mean±SD (n = 6) of three independent experiments.^#^*indicates significant differences with the respective control (^#^2.8 or *16.7 mM glucose) (Two-way ANOVA, Dunnett's post-hoc, p<0.05).

Direct or acute effects of the pollutants bisphenol A (BPA) (1 nM, 10 nM and 100 nM) and bis(2-ethylhexyl)phthalate (DEHP) (1 µM, 10 µM and 100 µM) on insulin secretion were assessed, using soluble, non-cytotoxic concentrations (data not shown). IBMX (50 µM) and diazoxide (100 µM) were included in this experiment as positive and negative controls for GSIS, respectively. As expected, diazoxide decreased GSIS significantly (p<0.0001), but for IBMX significant stimulation of GSIS was absent, though tending to stimulation (p = 0.072) (Fig. 3). Neither BPA, nor DEHP affected GSIS. BIS was however significantly increased by both compounds: a 30% increase by 100 µM DEHP (1.02 ± 0.11 ng insulin*100,000 cells^−1^), and a dose-dependent increase by BPA of ≈ 30% at 10 nM (1.05±0.14 ng insulin*100,000 cells^−1^) and ≈ 60% at 100 nM (1.23 ± 0.17 ng insulin*100,000 cells^−1^) compared to solvent control (0.76 ± 0.15 ng insulin*100,000 cells^−1^) (Fig. 3).

### Chronic effects of pharmaceutical compounds: building a framework for screening of chronic pollutant effects

**Figure 4:**
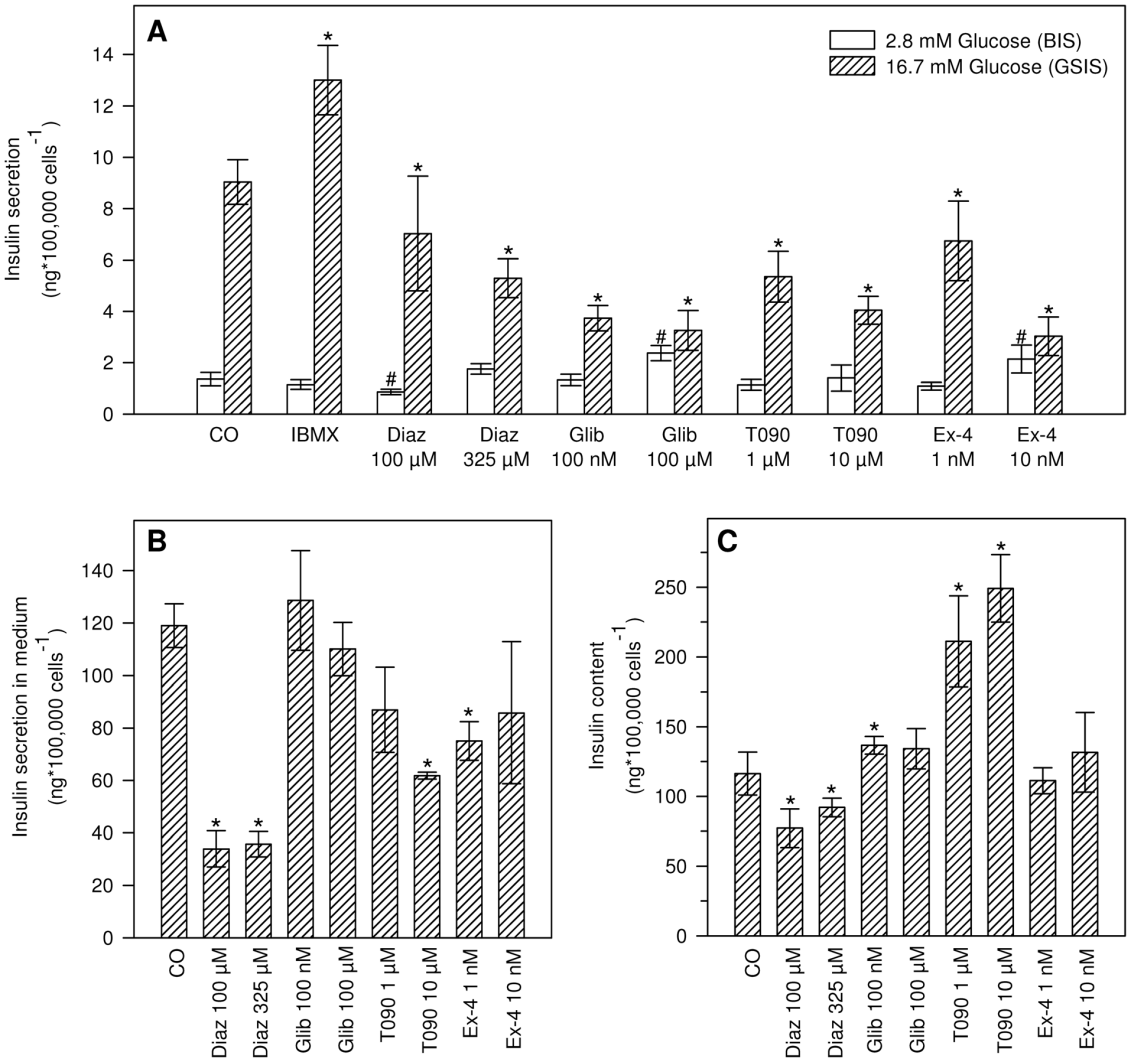
Chronic (72 h) effects of selected pharmaceuticals on insulin secretion and insulin content. (A) Insulin secretion after chronic (72 h) exposure to the indicated pharmaceuticals. Insulin secretion response was measured at 2.8 mM glucose (white bars) and 16.7 mM glucose (shaded bars) (n = 6). #*indicates significant differences with the respective control (#2.8 or *16.7 mM glucose) (Two-way ANOVA, Dunnett's post-hoc, p<0.05) (B) 24 h insulin secretion in standard culture medium during last 24 h of 72 h exposure period (n = 3). *indicates significant differences with solvent control (Two-way ANOVA, Dunnett's post-hoc, p<0.05) (C) Total insulin content after 72 h exposure to the indicated pharmaceuticals (n = 6). *represents significant differences with the solvent control (Two-way ANOVA, Dunnett's post-hoc, p<0.05). Data represent the mean ± SD of three independent experiments.

With regard to chronic effects of pharmaceuticals on insulin secretion, Figure 4A shows that all compounds significantly decreased GSIS following exposure, with exception of IBMX (positive control of GSIS). Glibenclamide (100 µM) and exendin-4 (10 nM) stimulated basal insulin secretion, while chronic exposure to 100 µM diazoxide resulted in decreased secretion at 2.8 mM glucose. Measurement of insulin secretion in RPMI 1640 with standard 11 mM glucose culture conditions during the last 24 h of the 72 h exposure indicated that both concentrations of diazoxide, 10 µM T0901317 and 1 nM exendin-4 decreased long term insulin secretion (Fig. 4B). Total insulin content after 72 h of exposure to diazoxide significantly decreased and T0901317 significantly increased insulin content (Fig. 4C). Glibenclamide did not alter 24 h insulin secretion (Fig. 4B), slightly augmented insulin content (100 nM; Fig. 4C) and severely affected insulin secretion in the acute secretion assay (Fig. 4A).

## Discussion

The hypothesis of metabolic disruptors is gaining momentum [5], [28] and recent reports have emphasized the importance of determining the biological plausibility of pollutants affecting processes leading to metabolic disease development [17]. As beta-cell function is one of the main determinants for the development of overt type 2 diabetes, appraisal of the diabetogenic metabolic disruptor capacity should rely on endpoints integrating aspects of beta-cell function. Therefore, development and use of reliable, cost-effective and physiologically relevant beta-cell based screening assays, which mimic primary beta-cell physiology as close as possible is essential. In the present paper, we evaluated if the INS-1 832/13 cell line could be used as a beta-cell based system to assess pollutant effects on insulin secretion.

### Optimization of exposure conditions maximizing glucose stimulated insulin secretion

To assure robust glucose stimulated insulin secretion, an absolute requirement for screening purposes, optimization of exposure conditions and experimental set up was emphasized in a first step, focusing purely on glucose sensitivity and secretory responses. Because it has been shown before that cell-cell contact influences insulin secretion [29], seeding density and exposure length, directly related to the cell number at the time of secretion assays, inherently influence beta-cell functionality and thus are important aspects to optimize. Our experiments showed that the insulin secretion index reaches a plateau phase at a final density of approximately 1,250,000 cells/well (Fig. 1B). Since some compounds were reported to affect beta-cell mass (e.g. exendin-4 [30], DEHP [10]) during chronic exposures, they might affect insulin secretion without effectively altering beta-cell function. Because this study aimed to specifically develop a system to screen effects on beta-cell function, further experiments were performed in a set up in which a density of 1,250,000 cells/well was reached at the time insulin secretion was assessed. In this way slight changes in cell density due to compound exposures are not reflected at the ISI level.

### Acute effects of pharmaceutical compounds: selection of positive and negative controls for insulin secretion assays

As some pollutants have the potency to directly affect insulin secretion (reviewed in [6]), evaluation of acute compound-induced changes in insulin secretion was further optimized in this cell system (Fig. 2). In this perspective, selection of appropriate reference compounds, i.e. positive and negative controls, is crucial. Lack of ill-defined beta-cell function modifying pollutants forces use of pharmaceuticals as control compounds.

Of the tested chemicals, IBMX was the strongest potentiator of GSIS resulting in an increase by about 75% (Fig. 2), while exendin-4, a glucagon-like peptide-1 (GLP-1) receptor binding compound tended to stimulate GSIS (≈ 30%), though non-significantly. Neither IBMX, nor 1 nM exendin-4 stimulated BIS, but 10 nM exendin-4 did, conform previous observations [31]. For both compounds, GSIS results are in agreement with previous observations and, based on the amount of induction, IBMX appeared to be the most suited positive control for secretion assays in our system.

Glibenclamide (100 nM), a sulfonylurea compound which binds and closes ATP-sensitive potassium channels (K_ATP_-channels) and thereby stimulates insulin secretion, did not affect GSIS in our set up. This was not surprising since Hohmeier et al. (2000) [23] already reported non-significant GSIS stimulation by the sulfonylurea tolbutamide in INS-1 832/13 cells. The reported increase of BIS by sulfonylurea's [32] is however confirmed by our results. Because INS-1 832/13 cells are highly responsive to glucose, it is likely that the sensitivity of K_ATP_-channels towards glibenclamide is reduced, rather than being the consequence of dysfunctionality of the K_ATP_-channels. Additional proof of the presence of fully operational K_ATP_-channels is not only provided by the stimulatory properties of glucose, but also by the dramatic decrease in secretion imposed by diazoxide, a compound that also targets K_ATP_-channels, but opens them instead of closing. Diazoxide clearly and dose-dependently reduced insulin secretion at 16.7 mM glucose, and thus can be selected as a negative control during insulin secretion assays.

The far less studied T0901317, an agonist of the nuclear receptor Liver X Receptor (LXR), was included in the analysis of potential positive and negative control compounds because many known endocrine disruptors (of which metabolic disruptors are a subclass) also exert their actions by binding to nuclear receptors. As such, it serves as a good reference compound to reflect the potential mode of action of metabolic disruptors in beta-cells. It has been reported that pancreatic islets, exposed acutely to 1 µM T0901317 do not show altered responses to glucose [33], which is confirmed by our results in INS-1 832/13 cells.

In general, these results show that INS-1 832/13 cells respond relatively normal in acute exposure scenario's to known beta-cell reactive compounds, confirming its status as relevant beta-cell model for studying acute insulin secretion responses. It should be noted, however, that only the lowest concentrations used, revealed “normal” profiles. One possible explanation might be subtoxicity of the highest concentrations, not detected with our robust cytotoxicity assay.

### Acute effects of pollutants: bisphenol A and bis(2-ethylhexyl)phthalate

To get a first indication of the relevance of INS-1 832/13 cells in a pollutant screening context the direct, acute effects of two human-relevant pollutants, bisphenol A and bis(2-ethylhexyl)phthalate on insulin secretion were investigated.

The plasticizer bisphenol A (BPA) has been extensively studied with regard to its effects on beta-cell function and the general consensus on BPA action on beta-cells is that it increases insulin content (chronically) [34], [35] and augments glucose stimulated insulin secretion (both acutely and chronically) [19], [35]-[37]. Compared to studies using similar BPA doses (1 nM and 10 nM) [36], our results on the acute effect of BPA on INS-1 832/13 cells show major differences. For example, in mice islets no acute effect of 1 nM BPA on BIS was observed [36], while GSIS was significantly potentiated (at 8 mM glucose in [36]). Similar results were obtained for BPA exposed human islets, though effects on BIS were not reported [36]. For higher concentrations of BPA (438 nM, 4.38 µM) Makaji et al. (2011) [19] report increased BIS in beta-TC-6 cells conform our results in INS-1 832/13 cells, but they also observed increased GSIS, absent for the INS-1 cell system. It should be noted however that, besides the disparity in effective dose (i.e. 10 nM and 100 nM BPA in our study; 438 nM and 4.38 µM in [19]), some major differences in glucose response profiles exist between both beta-cell models which may explain the different observations. For instance, beta-TC-6 cells are far more sensitive to glucose with a half maximal stimulatory glucose concentration of 0.5 mM glucose [21], whereas this is 6 mM glucose for INS-1 832/13 cells [23]. Therefore, the observed results for BIS (obtained at 3.3 mM glucose) in beta-TC-6 cells may represent effects on GSIS, rather than BIS.

It should be emphasized that, in agreement with our results for the rat INS-1 832/13 cell line, previous experiments on isolated rat islets also showed no effect of BPA (0.438 nM – 4.38 µM) on GSIS (at 8.3 mM and 16.7 mM glucose) [40]. Effects on BIS in rat islets were not mentioned. As such, the absence of acute changes in GSIS by BPA may be species specific, further endorsed by the fact that some major species differences in responses to glucose stimulation have been reported [41]. Furthermore, since direct stimulatory effects of BPA on insulin secretion are suggested to be partly mediated by estrogen receptor (ER) beta binding, (amongst others), absence or insensitivity of ERs in INS-1 cells might explain lack of stimulatory effects on GSIS. Indeed, Horn et al. (2000) [38] reported that INS-1 cells, from which the INS-1 832/13 clone is derived, only marginally express ERs. Conversely, INS-1 cells have been used in a recent study [39] to explore suppressive effects of 17beta-estradiol via ERalpha, ERbeta and G-protein coupled ER on lipogenesis, obviously assuming presence of operational ERs. Whether or not lack of effect of BPA on GSIS in INS-1 832/13 cells is the consequence of differences in ER expression patterns remains to be determined.

For bis(2-ethylhexyl)phthalate (DEHP), only information on developmental exposure effects on beta-cell function is available. It has been shown that in female offspring from DEHP-exposed rats both beta-cell mass, beta-cell area and insulin content decreased, combined with degranulation [10]. Furthermore, in vivo and ex vivo responses to a glucose stimulus declined and first phase insulin secretion was impaired [10]. Direct effects of DEHP on insulin secretion, both acute and chronic, have not been reported so far. Nevertheless, because DEHP is known to act in other tissues via peroxisome proliferator-activated receptors (PPARs) [42] and since PPARs are suspected to participate in processes related to beta-cell function, both acutely and chronically [43], [44], it was valuable to include DEHP in the initial evaluation of INS-1 832/13 to screen pollutants for diabetogenic properties. In our experiments, DEHP only affected BIS at a relatively high concentration (100 µM) and thus appears to lack effects at environmentally relevant doses in INS-1 832/13 cells.

Although our results on acute BPA effects oppose previous reports, which may indicate limited applicability of INS-1 832/13 cells as a screening tool for acute pollutant effects, examination of chronic effects are necessary to allow a comprehensive evaluation. Foremost, though well defined for mice islets, no acute effects of BPA have been found in isolated rat islets [40] and only chronic effects have been reported [35], [40]. In addition, DEHP is well known to bind PPARs, nuclear receptors that affect insulin secretion when activated chronically [43]. The next step in this evaluation procedure was thus focused on creating a framework to enable chronic exposures to environmental pollutants.

### Chronic effects of pharmaceutical compounds: building a framework for screening of chronic pollutant effects

In building this framework, we firstly investigated whether INS-1 832/13 cells are a relevant system to study chronic responses using acknowledged chronic beta-cell function stimulators or inhibitors. In general, none of the tested pharmaceuticals behaved as expected based on chronic, albeit relatively scarce, literature data (Table 1, 2). For some compounds, one or two of the tested insulin secretion related endpoints were in accordance with known effects. For instance T0901317 increased insulin content [33], [45], exendin-4 left insulin stores unaltered [32], and for exendin-4 (GLP-1 agonist) the previously reported stimulatory effect on INS-1 832/13 growth was also observed as indicated by significantly increased cell numbers (data not shown) [46]. However, none of the tested compounds entirely fitted the suspected profile.

**Table 2:** Overview of chronic effects of selected pharmaceuticals on beta-cell function as previously described and the results obtained in this study.

Compound	Literature					Present study				
	BIS	GSIS	C	M	Test conditions	BIS	GSIS	C	M	Test conditions
Diazoxide	↑	↑	↑	↓	24 h; 325 µM; rat islets [49]	↓	↓	↓	↓	72 h; 100 µM; INS-1 832/13
	=	↑	↑	↓	24 h; 325 µM; rat islets [50]	=	↓	↓	↓	72 h; 325 µM; INS-1 832/13
	=	=	=	↓	24 h; 100 µM; INS-1E [51]					
Glibenclamide	↓	↓	↓	NA	72 h; 100 µM; BRIN-BD11 [53]	=	↓	↑	=	72 h; 100 nM; INS-1 832/13
	↑	↓	↓	NA	18–20 h; 10 nM; mice islets [54]	↑	↓	=	=	72 h; 100 µM; INS-1 832/13
					24 h; 100 nM; mice islets [55]					
	↑	↓	↓	NA	24 h; 10 µM; human islets [56]					
T0901317	=	↑	↑	NA	72 h; 1 µM; rat islets [33]	=	↓	↑	=	72 h; 1 µM; INS-1 832/13
	↓	↑	↑	NA	72 h; 1 µM; MIN6 [33]	=	↓	↑	↓	72 h; 10 µM; INS-1 832/13
	↑	↑	↑	NA	72 h; 1 µM; INS-1E [45]					
Exendin-4	=	↑	=	NA	16h; 10 nM; rat islets [32]	=	↓	=	↓	72 h; 1 nM; INS-1 832/13
	=	↑	↑	NA	72 h; 10 nM; human islets; GLP-1 [58]	↑	↓	=	=	72 h; 10 nM; INS-1 832/13

Data on basal insulin secretion (BIS), glucose stimulated insulin secretion (GSIS), insulin content (C) and insulin secreted in culture medium (M) are given.

Thus, although the cells still showed appropriate glucose and IBMX responsiveness (Fig. 5A), the biology of INS-1 832/13 cells appears to fundamentally differ from pancreatic islets and other cell lines with regard to their chronic response to control compounds. As such, generation of a framework for chronic pollutant testing and ranking is hampered. Moreover, these results discouraged further testing of chronic BPA and DEHP effects and are not supportive for future INS-1 832/13 based pollutant screening.

### Defects in chronic responses of INS-1 832/13 cells: explanations and alternatives

A possible explanation for inappropriate responses of the INS-1 832/13 cell line to chronic stimuli might lay within overstimulation of the cells due to repeated medium replacement. This renders them more sensitive to glucose (Fig. 1B), but might push them to the limits of secretory capacity and might induces loss of sensitivity for other stimulators (e.g. IBMX is not always a significant stimulator (Fig. 3)). However, preliminary experiments with cells which were cultured differently gave comparable results for chronic and acute exposures to the selected pharmaceuticals, although they were much less glucose responsive (ISI of 6 versus 14 in the present research) in the control condition using the same set up (data not shown). Thus although cells are pushed to their limits in the proposed set up, this is not likely to be the main cause for absence of effects.

Another interfering factor that might compromise interpretation of long term exposures is constitutive expression of the inserted human proinsulin gene in INS-1 832/13 cells [23]. Especially if the main impact is suspected to occur at the level of transcriptional regulation, effects on insulin secretion and insulin content might be missed in INS-1 832/13 cells. An alternative approach would be to select representative beta-cell lines which were not generated by genetic manipulation, such as the clonal INS-1E system [47].

Although testing INS-1E cells in the framework of metabolic disruptor screening is a future perspective, another issue arises when using only cell lines for assessing long term metabolic disruptor effects. Endocrine disrupting compounds mainly operate via nuclear receptor (des)activation. For INS-1 cells, opposing reports regarding nuclear receptor expression (e.g. estrogen receptor expression) [38], [39] may be explained by the heterogenic nature of this cell population [47]. However, comparisons between other cell lines (e.g. MIN6 cells) and primary beta-cells have also revealed substantial differences in nuclear receptor expression profiles [48]. Therefore, in depth characterization of nuclear receptor expression profiles in all existing cell lines in comparison with primary beta-cells is urgent and required, before any (chronic) pollutant testing initiative can be started.

In conclusion, although efforts are being made to evaluate the applicability of existing beta-cell lines, based on the information presently available primary beta-cells seem the best alternative for screening of acute and certainly chronic pollutant effects.

### Conclusions

The present paper evaluated whether the INS-1 832/12 cell line could be used as a cell based system to screen the diabetogenic potential of environmental pollutants. Although our analyses confirmed that INS-1 832/13 cells are highly sensitive to glucose, show high insulin secretion capacity, and respond well to known potentiators and inhibitors of insulin secretion in acute conditions, we also revealed that responses to known beta-cell function disrupting pollutants (BPA and DEHP) showed some deviations compared to previously reported results in other cell lines, primary beta-cells or isolated islets. Furthermore, chronic effects of pharmaceutical compounds on insulin content and secretion greatly differed from past observations. Although detailed mechanistic research is needed to answer the question why these deviations occur, the INS-1 832/13 cell line appears to lack certain characteristics needed to respond appropriately to either acute pollutant exposure, either long term effects of pharmaceuticals. As such, with the current knowledge at hand, INS-1 832/13 cells seem inadequate as a diabetogenic pollutant screening system.
